# Chemical doping of unsubstituted perylene diimide to create radical anions with enhanced stability and tunable photothermal conversion efficiency

**DOI:** 10.3389/fchem.2023.1187378

**Published:** 2023-04-25

**Authors:** Canyan Che, Shaohua Tong, Yanhua Jia, Jiaji Yang, Xiandong He, Shaobo Han, Qinglin Jiang, Yuguang Ma

**Affiliations:** ^1^ State Key Laboratory of Luminescent Materials and Devices, Institute of Polymer Optoelectronic Materials and Devices, South China University of Technology, Guangzhou, China; ^2^ School of Textile Materials and Engineering, Wuyi University, Jiangmen, China

**Keywords:** controllable doping, perylene diimide, radical anion, stability, photothermal conversion

## Abstract

N-doping of perylene diimides (PDIs) to create stable radical anions is significant for harvesting photothermal energy due to their intensive absorption in the near-infrared (NIR) region and non-fluorescence. In this work, a facile and straightforward method has been developed to control the doping of perylene diimide to create radical anions using organic polymer polyethyleneimine (PEI) as a dopant. It was demonstrated that PEI is an effective polymer-reducing agent for the n-doping of PDI toward the controllable generation of radical anions. In addition to the doping process, PEI could suppress the self-assembly aggregation and improve the stability of PDI radical anions. Tunable NIR photothermal conversion efficiency (maximum 47.9%) was also obtained from the radical-anion-rich PDI-PEI composites. This research provides a new strategy to tune the doping level of unsubstituted semiconductor molecules for varying yields of radical anions, suppressing aggregation, improving stability, and obtaining the highest radical anion-based performance.

## 1 Introduction

Perylene diimide (PDI) and its derivatives (PDIs) are among the most popular organic dyes and pigments. They are also excellent n-type organic semiconductors with high molecular absorption coefficients, high electronic affinity, high electron mobility, and good photothermal stability (Hu et al., 2021). Radical anions or dianions of PDIs can be easily generated *via* one-electron transfer or two-electron transfer pathways under chemical, electrochemical, or light/thermal-induced reduction. PDI radical anions feature intensive NIR light absorption and photo-energy conversion into heat *via* non-radiative relaxation pathways such as molecular vibrations (Jiao et al., 2015; Yang et al., 2017; Zhang et al., 2018; Yang and Chen, 2019), which makes them promising photothermal materials. Thanks to these features, PDIs’ radical anions are widely used as optoelectronic materials in the fields of solar cells (Zhao et al., 2018; Yao et al., 2020; Hu et al., 2021), transistors (Schmidt et al., 2009; Gao and Hu, 2014; Okamoto et al., 2020), electrochromic devices (Ma et al., 2016), thermoelectrics (Russ et al., 2014; Wu et al., 2017), photocatalysis (Ghosh et al., 2014; Gong et al., 2018; Zhang et al., 2020), and redox flow batteries (Li et al., 2018). Among the large family of PDIs, unsubstituted PDI is the simplest model molecule that is commercially available and inexpensive. However, it is rarely used directly as a photothermal or an organic optoelectronic material due to the instability of its reduced species and strong aggregation of PDI, which result in phase separation during solution processing.

Functionalization is a fundamental strategy used to suppress aggregation, improve solubility, and enhance the stability of reduced molecules (Gao et al., 2014; Zhou et al., 2019; Golshan et al., 2020). The amino-functionalized PDIs have been demonstrated as an especially effective self-n-doping system with enhanced doping capability under mild conditions, according to Powell and Whittaker-Brooks (Powell et al., 2022; Powell and Whittaker-Brooks, 2022). However, molecular functionalization not only requires complex synthesis procedures but also reduces the content of PDI chromophore in the overall molecular weight and elicits a risk of side effects. For example, the resulting functionalized PDIs normally have a larger torsion of the planar backbone and changes in their electronic structure, such as less compact π-π stacking, which is crucial for the intermolecular charge transport along the one-dimensional (1D) π-stacking direction (Chen et al., 2007) and novel properties such as room temperature ferromagnetism (Jiang et al., 2022). Therefore, it is necessary to avoid substituents when exploring the inherent photothermal, magnetic, and optoelectronic properties of the PDI molecule itself (Dong et al., 2020).

However, the inferior solution processability and strong aggregation of neutral and n-doped PDI need to be improved. To track those issues, our group first reported an ionization strategy that employs hydrazine hydrate as a solvent and a strong reducing agent to dissolve and reduce PDI (Jiang et al., 2020; Jiang et al., 2022). As a result, a homogenous mixture of PDI and hydrazine hydrate can be obtained, and the suspension is feasible for film casting. However, only the PDI dianion could be obtained directly after the reduction process because of the strong reducing ability and the excess amount of hydrazine hydrate. PDI radical anions were produced by subsequent spontaneous oxidation of the dry film in the air.

This method has two drawbacks. First, it is a challenge to precisely control the de-doping process of dianions to form anions that can be oxidized to neutral PDI in the following spontaneous oxidation process. Second, it was risky to use the strongly carcinogenic hydrazine hydrate under high temperatures and high pressure during the hydrothermal reaction. Therefore, diluting strong reducing agents or using mild reducing agents to obtain radical anions directly is significant for the controllable generation of PDI radical anions. In another aspect, using a cationic reducing polymer is also conducive to improving the stability of PDI radical anions *via* physical encapsulation and electrostatic interaction. For example, the positively polarized moiety, cucurbit [7]uril (CB [7]), was reported to suppress the quenching of PDI radical anions *via* a supramolecular strategy (Jiao et al., 2015), and tetra cationic cyclophane (ExBox4^+^) was found to improve the stability of radical anions *via* the surrounding Coulomb attraction and encapsulation (Jiao et al., 2019).

Polyethylenimine (PEI (C_2_H_5_N)_n_) with a large number of amine groups is a mild and non-toxic polymer reducing agent that has been used widely in the reduction of noble metal nanoparticles and preparation of corresponding core-shell nanocomposites, such as Au and Ag (Zhang et al., 2013; Mulens-Arias et al., 2019; Demchenko et al., 2020). It has been proved that PEI is an effective doping agent for various organic and inorganic semiconducting materials, such as fullerene derivative (PCBM) (Dong et al., 2016), poly(benzimidazobenzophenanthroline) (BBL) (Yang et al., 2021), naphthalene diimide derivative (P(NDI2OD-T2)) (Long et al., 2017), polythiophene derivatives (PDBTAZ, PPzDPDP-BT, PDBPyBT, and PDQT) (Sun et al., 2015), carbon nanotubes (Rdest and Janas, 2021), graphite oxide (Tadjenant et al., 2020), and MoS_2_ (Hong et al., 2017). In addition, the cationic polyelectrolyte also helps to improve the stability of reduced products *via* physical encapsulation and strong electrostatic attraction (Remant Bahadur and Uludağ, 2016). However, there is as yet no report on the doping of PEI on unsubstituted PDI or on the effect of PEI in suppressing the aggregation and stabilization of PDI radical anions.

Here, the polymer reducing agent PEI was employed for preparing PDI-PEI composites with varying doping intensity to explore new strategies for controllable doping of PDI and investigate the effect of dopants on aggregation and stability. UV-vis spectroscopy was used to characterize the doping evolution of PDI in the composite with increasing amounts of PEI and to reveal the effect of PEI on suppressing the aggregation of PDI radical anions and monomers together with field emission scanning electron microscopy. Electron paramagnetic resonance was used to demonstrate the formation of radical anions, and dynamic light scattering measurement was carried out to study the particle size of the PDI-PEI composites. The photothermal conversion efficiency of the radical-rich PDI-PEI composites was also investigated based on the amount of radical anions with tunable yield in the composite.

## 2 Experimental section

### 2.1 PDI doping

To tune the doping degree of PDI-PEI composites, 0.04 mg/mL PDI (J&K Scientific) was dispersed in N, N-dimethylformamide (DMF, 99.9%, extra dry with molecular sieves, Innochem) with various amounts of PEI (branched, Mw = 600, Energy Chemical) content, then the suspensions were mixed and treated with an ultrasonic homogenizer at the power of 540 W (JY92-IIDN, Scientz), followed by incubation at 140°C for 24 h. The mass ratio of PEI to PDI is 0.5, 1, 2, 5, 10, 20, 50, 80, 100, 200, 300, 400, 500, 800, 1,000, 2000, and 3,000, which corresponds to a PEI repeat unit/PDI molar ratio of 4.5, 9.1, 18.1, 45.3, 72.6, 90.7, 181.3, 453.6, 725.7, 907.1, 1814.3, 4,535.8, 7,257.3, 9,071.6, 18,143.2, and 27,214.7.

### 2.2 Characterization

UV-vis absorption measurements of the series of reacted PDI-PEI suspensions were carried out with a demountable 2-mm quartz cuvette in a glovebox. UV-vis spectra of hydrazine-hydrate-reduced PDI and PEI-reduced PDI in DMF in exposure to air were conducted in an ambient atmosphere with quartz of diameter = 10 mm.

Electron paramagnetic resonance (EPR) was recorded on a Bruker E500 EPR spectrometer (300 K, 9.854 GHz, Xband, Karlsruhe, Germany). The microwave power was 6.325 mW, and the amplitude modulation was set to 1 G.

The twinning of PEI on PDI was demonstrated by microscopy and dynamic light scattering measurements. Microscopic images were obtained using a field-emission scanning electron microscope (SEM) (ZEISS, Oberkochen, Germany) at room temperature. The suspensions were deposited onto silicon, followed by drying at 60°C. The dry samples were gold coated for 30 s at 15 mA prior to fixing in the SEM holder for imaging. Dynamic light scattering (DLS, 90Plus PALS, Brookhaven Instruments Corporation) was used to measure the particle size of the PDI suspensions doped with varying PEI content.

The electronic level of PDI aggregates and monomer was estimated from cyclic voltammetry and spectroscopy. Cyclic voltammetry of pristine PDI with and without PEI and monomeric PDI with PEI was carried in a glass cell in a three-electrode system in a glovebox. Glassy carbon was used as the working electrode, Pt mesh was the counter electrode, and Ag/Ag^+^ was the reference electrode. The reference electrodes were calibrated with Fc/Fc^+^ before use. Tetrabutylammonium hexafluorophosphate (n-Bu_4_NPF_6_, 98%, J&K Scientific) was recrystallized from ethanol and dried at 120°C in an oven for 12 h before being dissolved in DMF and used as an electrolyte.

The LUMO energy level was calculated from the onset reduction potential in the voltammogram according to the following formula:

ELUMO=−4.8+EredPDI0−E12Fc/Fc+,

where $E_{red}^{{PDI}^{0}}$ is the onset reduction potential of the neutral PDI and $E_{1/2}^{Fc/{Fc}^{+}}$ is the redox potential of ferrocene/ferrocenium Fc/Fc^+^ ≈ 0.089 V vs. Ag/Ag^+^ in a DMF electrolyte with 0.1 M Bu_4_NPF_6_.

The HOMO energy level was obtained by

$$E\left(HOMO\right)=E\left(LUMO\right)-E_{gap},$$

where $E_{gap}$ is the energy gap calculated according to ∆E = 1,240 nm/λ_onset_ and λ_onset_ is the long wavelength edge of the absorption band.

Photothermal conversion data were collected by recording the temperature changes of 2 mL of suspension when the laser irradiation was on and off (Blueprint, VCL-808nmM0-2W). The power was set to 1.4 W, and the laser spot radius was 10 mm. The photothermal conversion efficiency, *η,* was calculated from equation $\eta =\frac{hs∆T-Q_{dis}}{I\left(1-10^{-A_{\lambda }}\right)}$ , where h is the heat transfer coefficient, s is the surface area of the container, ∆T is the temperature change of the PDI-PEI suspension at the maximum steady-state temperature, *I* is the laser power, $A_{\lambda }$ is the absorbance of the composite suspension at 808 nm, and $Q_{dis}$ is the heat associated with the light absorbance of the solvent.

Magnetization was measured using a Quantum Design PPMS-9 with a vibrating sample magnetometer at room temperature. The diamagnetic correction was performed using diamagnetic susceptibility from the sample holder.

## 3 Results and discussion

**FIGURE 1:**
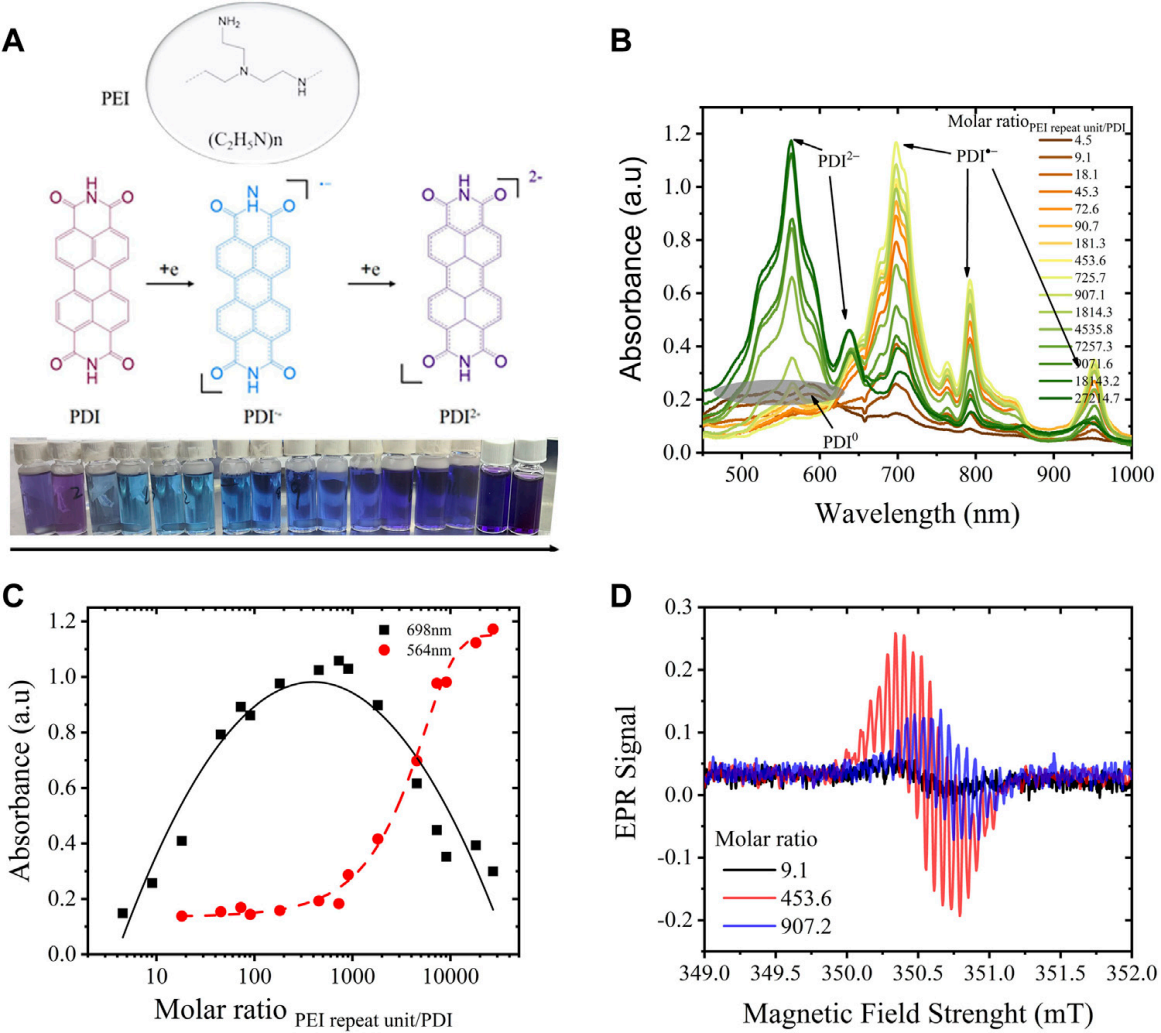
**(A)** Chemical doping of PDI (0.04 mg/mL in DMF) by various amounts of PEI to form radical anions/dianions; **(B)** UV-vis spectra of the reduced PDI-PEI suspensions, the molar ratio of PEI repeat unit/PDI increase from 4.5 to 27215; **(C)** a plot of the intensity of absorption at 698 nm, 564 nm assigned to PDI-and PDI2-, vs molar ratio of PEI repeat unit/PDI; **(D)** Electron paramagnetic resonance spectra of PDI suspension doped with PEI content under a molar ratio of PEI repeat unit to PDI equals to 9.1, 453.6 and 907.2.

The PDI-PEI composite suspension was first prepared by the one-pot synthesis described in the experimental section. As shown in Figure 1, a color change from fuchsia to blue and purple can be recognized by the naked eye from the PDI suspensions with increasing content of PEI, visualizing the varied doping extent of PDI-PEI composite caused by the electron transfer from PEI to PDI. To define the PDI changes, UV-vis spectra of raw PDI and PDI monomer (depolymerized electrochemically) were collected (Supplementary Figure S1). The raw PDI suspension (0.04 mg/mL in DMF, treated with 5 min of high-power sonication) showed a broad UV-vis absorption around 480–550 nm and a new absorption around 590 nm due to aggregation. The neutral monomeric PDI (depolymerized electrochemically) showed a characteristic absorption around 453 nm, 486 nm, and 521 nm, denoted to their (0,2) (0,1), and (0,0) electronic transitions, close to the reported neutral PDI derivatives that featured main absorptions at 458 nm, 490 nm, and 526 nm, respectively (Golshan et al., 2020). The PDI radical was characterized by main absorptions at 707 nm, 797 nm, and 961 nm, and PDI dianions displayed absorptions at 566 nm and 637 nm. The chemically doped PDI is characterized by similar absorptions. With the gradual increase of PEI content (Figures 1B,C), absorption peaks around 698 nm, 791 nm, and 951 nm emerged and increased while the broad absorption around 480–550 nm decreased. This shift was assigned to the process of PDI aggregates being chemically reduced to radical anions ( $PDI\overset{e^{-}}{\to }{PDI}^{∙-}$ ). When the PEI repeat unit/PDI molar ratio increased to 725.7 (mass ratio of PEI/PDI = 80), the absorption of radical anions saturated, predicting a depletion of neutral PDI and the highest concentration of PDI radical anion (close to 0.04 mg/mL, that is, ∼1 electron/molecule on average). The absorption around 565 nm and 637 nm, assigned to the PDI dianion, arose slowly, far before the exhaustion of PDI radical anions, indicating that a small fraction of PDI experiences a chemical disproportionated reaction from a neutral state to dianion directly ( $PDI\overset{2e^{-}}{\to }{PDI}^{2-}$ ). The intensity of the PDI dianion absorption continued to increase dramatically when more PEI was added, and the concentration of PDI radical anion started to decrease, corresponding to the process of ${PDI}^{∙-}\overset{e^{-}}{\to }{PDI}^{2-}$ . When the PEI repeat unit/PDI molar ratio increased to 27,214 (or a mass ratio of PEI/PDI = 3,000), the UV-vis absorptions around 698 nm, 791 nm, and 951 nm were comparable to those of the suspensions, which had a PEI repeat unit/PDI molar ratio of 9.1 and 18.1, suggesting a similar fraction of PDI^∙−^ but a majority fraction of PDI^2−^ instead of PDI. The largest yield of PDI dianions (∼2 electrons/molecule on average) might be obtained if PEI content were increased further. Therefore, the doping level of PDI and the yield of PDI radical anions can be controlled precisely in a wide range with varying content of PEI. The maximum yield of radical anions was a little smaller than 100% due to the disproportional reaction of the neutral to the dianion.

DLS results of PDI-PEI composite in suspension exhibited a positive correlation of hydrodynamic diameter of PDI-PEI particles with PEI content in DMF. As shown in Supplementary Figure S2, the hydrodynamic diameter of the composite increased from 122 nm to 867 nm with an increase in PEI content (the PEI repeat unit/PDI molar ratio increased from 4.5 to 90.7), where neutral PDI was consumed to generate the radical anions. The highest amount of radical anion was created with a PEI repeat unit/PDI molar ratio of 725.7, as illustrated in Figure 1C. The particle size dependence in this range visualized the successful bonding of PEI to the PDI radical anion. The diameter plateau of PDI-PEI particles is around 1 μm. More PDI radical anions were expected to be generated when more PEI was added; the plateau suggests that an electrostatic and steric balance is reached on the PDI radical anion-PEI composite particles before the formation of the PDI dianion-PEI particles. The paramagnetic radical anion caused by electron transfer from PEI to PDI was also demonstrated by electron paramagnetic resonance spectroscopy (Figure 1D), with a well-resolved hyperfine splitting pattern and a g-factor value of 2.00305 (Supplementary Figure S3). Two equivalent N atoms (at imide rings) and two distinct sets of four core-H atoms of PDI^∙−^ (H_a_ and H_b_) were responsible for the splitting pattern, confirming that electron density is mostly delocalized within the conjugated π-system (Goodson et al., 2013). Even though the intensity of the radicals differs and depends on concentration correlated with the doping state of PDI suspension, a symmetrical pattern was observed in all EPR signals, suggesting a similar delocalization degree of the electrons. SEM images demonstrated morphological changes of self-assembly PDI in the absence and presence of PEI.

**FIGURE 2:**
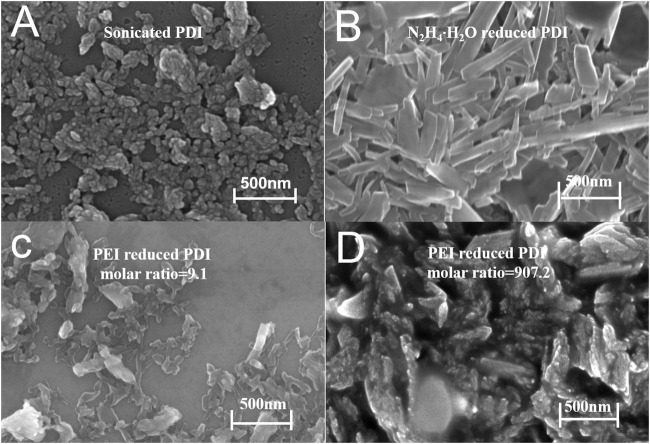
SEM images of sonicated PDI **(A)**; hydrazine hydrate reduced PDI **(B)**, and PEI reduced PDI with a molar ratio of PEI repeats unit/PDI equals 9.1 **(C)** and 907.2 **(D)**.

As shown in Figure 2, PDI aggregates can be dispersed into particles around 500 nm after sonication and cast on the silicon substrate. The hydrazine-hydrate-reduced PDI formed nanorods of 50–60 nm width and around 1 μm length by self-assembly of its dianions. A small amount of PEI in the composite (molar ratio of PEI repeat unit/PDI = 9.1) could lead to irregular debris shapes and clusters when the ratio is raised to 907.2. More PEI twinned onto PDI leads to a much more irregular cluster morphology, demonstrating that the presence of PEI in the solution suppressed the self-assembly of reduced PDI to form regular compact rods driven by π-π interactions (Jiang et al., 2022). This suppression of PEI in the self-assembly of the reduced PDI has also been visualized in the decreased ferromagnetism (Supplementary Figure S4).

**FIGURE 3:**
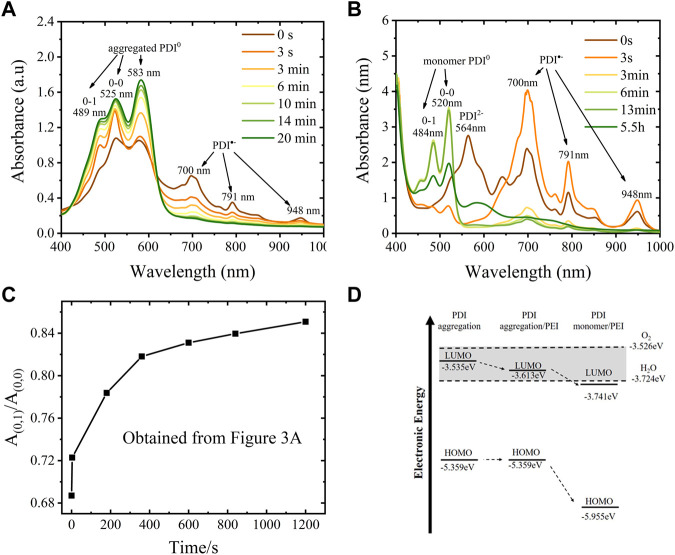
Stability of PDI radical anion and dianion using hydrazine hydrate and PEI as a dopant. **(A)** UV-vis spectra of hydrazine hydrate reduced PDI suspension that exposure to air or different time, 50 µL of the reduced suspension(10 mg/mL) was dropped cast on a clean glass substrate and dried at 120°C for 3 hours and then kept at 50°C overnight to remove excess hydrazine hydrate, then the dry film was scraped to dissolve in 5 ml DMF with a dropper in the glovebox before UV-vis spectroscopy, PDI concentration in DMF is ∼0.1 mg/mL; **(B)** UV-vis spectra of PEI reduced PDI suspension that exposure to air for a different time; **(C)** A plot of A_0–1_/A_0–0_ ratio as a function of time exposed to air, the data were obtained from Figure 3A; **(D)** Diagram of the calculated HOMO and LUMO energy of PDI monomer in DMF with or without PEI. The dashed lines are the LUMO energy levels of oxygen and water.

The effect of PEI in suppressing self-assembly aggregation of neutral PDI was also demonstrated by the UV-vis spectra presented in Figure 3. When the hydrazine-hydrate-reduced PDI was exposed to air, the absorption intensity of typical neutral PDI aggregates at 583 nm increased immediately due to the PDI radical anions being oxidized and becoming neutral aggregates, demonstrating a quick self-assembly-driven aggregation process of neutral PDI without PEI. The aggregation of PDI monomer is observed in hours in the presence of PEI. To be specific, the aggregation was identified to start after 5.5 h, accompanied by the appearance of the new peak around 583 nm, while the amount of neutral PDI monomer (485 nm, 520 nm) continued to increase for at least 13 min due to more PDI radical anions being oxidized into neutral PDI.

The twinning of PEI onto PDI not only limits the aggregating of PDI but also hinders the electron transfer between PDI dianion or radical/PEI composite and the oxidants, which can also improve the stability of PDI radical anions in the air due to physical encapsulation and electrostatic attraction. The UV-vis spectroscopy (Figure 3) illustrates the changes in ambient stability of PDI dianions and radial anions with or without PEI. The absorption peak of the PDI dianion in DMF solvent obtained by dissolving hydrazine-hydrate-reduced-film disappeared from the spectra, demonstrating that the PDI dianion is extremely unstable in DMF with little trace of oxide species as the DMF solvent and containers were deoxygenated overnight in a glovebox where the content of water was below 0.01 ppm, and oxygen was below 5 ppm. When exposed to the air for a longer time, the absorption of radical anions decreased gradually and disappeared in 20 min, while that of aggregated neutral PDI increased continuously. The maximum absorption ratio for the 0–1 vibronic transition (A_0–1_) and the absorption maxima of the 0–0 vibronic transition A_0–0_, that is, A_0–1_/A_0–0_ was observed to increase from 0.69 to 0.85, as shown in Figure 3C, indicating a more proximal packing of neutral PDI at higher monomer concentration according to Xin Wang (Wang et al., 2017) and Benjamin Fimmel et al. (Fimmel et al., 2015). Differently, the PEI-reduced PDI dianion and anion suspension in DMF is relatively stable in the same inert atmosphere and exists for more than a year when stored in a glovebox. However, the PEI-reduced PDI dianion was also very reactive in the air; its main absorption at 565 nm disappeared immediately in 3 s upon exposure to air. The main absorptions of the PDI radical anion at 700 nm, 791 nm, and 959 nm increased dramatically due to the spontaneous oxidation process of ${{PDI}^{2-}\overset{e^{-}}{\to }PDI}^{∙-}$ and then decreased gradually due to the following oxidation of ${PDI}^{∙-}\overset{e^{-}}{\to }\;PDI$ . The radical anion was more stable than the PDI dianion, illustrated by the existing small absorption peaks at 700 nm and 791 nm after 13 min (radical anion monomer) and the small broad absorption at 700–800 nm (radical anion aggregates) after 5.5 h.

The stability of the PDI radical anions in the presence of PEI in DMF solution (up to 13 min for its monomer and up to 5.5 h for its aggregates in contact with air) falls between the imide-position-substituted derivatives and the bay-substituted derivatives reported. For example, Marcon et al. reported self-assembled N, N′-Bis(2phosphonoethyl)-3, 4, 9, 10-perylenediimide radical anions reduced by sodium dithionite persisted for several minutes in the presence of oxygen (Marcon and Brochsztain, 2007). Schmidt et al. synthesized zwitterionic perylene diimide-centered radicals from cationic imidazolium-substituted tetrachloro-substituted perylene-3,4:9,10-tetracarboxylic acid bisimide (Pr_2_Im-PDI-Cl_4_). The lifetime of its solution in CH_2_Cl_2_ (1.35*10^–5^ M) was about 1 day, as revealed by UV-vis spectra (Schmidt et al., 2015). He et al. reported the bay-substituted perylene diimide radical anion (N, N-diethylhexyl-1,7-di (pentafluoro-phenoxyl) perylene diimide, DFPDI) was not only stable to moderate oxidants in ambient air (O_2_) and moisture for prolonged times but also was not sensitive to strong oxidants, acid H^+^, and strong oxidization metal ions with low limit values (He et al., 2016).

The LUMO-HOMO energy evaluated through voltammetry and UV-vis spectra shows that the electronic state of PDI is affected by PEI, as shown in Figure 3D and Supplementary Figure S5. The LUMO energy level of pristine PDI aggregation is about −3.535 eV, estimated from the onset reduction potential at −1.176 V in a cyclic voltammogram. The presence of PEI (50 mg/mL) in suspension leads to a more positive onset reduction potential of PDI aggregates at −1.098 V, while that of the PDI monomer in the presence of PEI shifted ∼0.128 V positively, indicating a lower LUMO energy level for PDI aggregation and PDI monomer in the presence of PEI (−3.613 eV and −3.741 eV, respectively). The corresponding HOMO energies were −5.359 eV, −5.359 eV, and −5.955 eV, respectively, for PDI aggregate without PEI, PDI aggregates in the presence of PEI, and PDI monomer in the presence of PEI when taking their energy gap (1.824 eV, 1.746 eV, and 2.214 eV, respectively) into account. Thus, both the HOMO and LUMO energy levels of PDI monomer in the presence of PEI were found to be lower than those of the PDI aggregation (Figure 3D). Note that the LUMO energy levels of ubiquitous oxidants (oxygen: 3.526 eV, water: 3.724 eV, Supplementary Figure S5C) are close to those of PDI. Lower LUMO and HOMO energy were normally considered to account for the promotion of electron injection and transportation (Gao et al., 2014) and the enhancement of the air stability of the reduction products. Promoted formation of radical anions with extraordinary stability was also reported by Song et al. on naphthalenediimides (NDIs), which had a lower LUMO energy and HOMO energy (Song et al., 2015). Therefore, the decrease of PDI’s LUMO energy (0.106 eV for PDI monomer) is important in improving the ambient stability of PDI radical anions.

**FIGURE 4:**
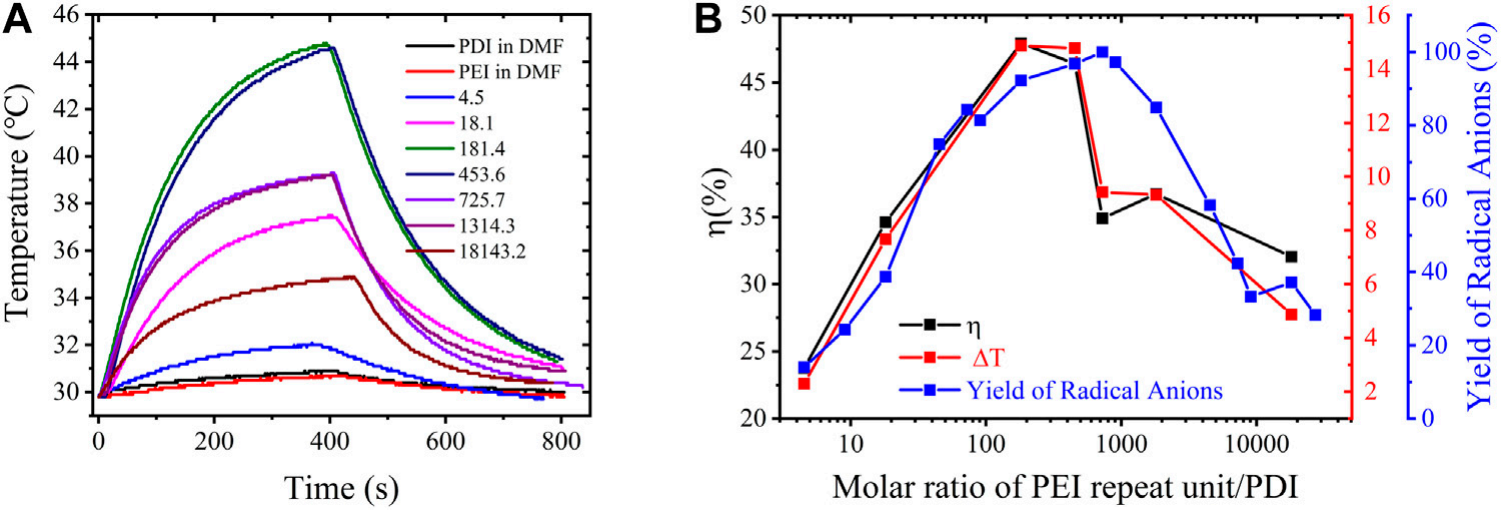
Photothermal conversion performance of PEI-PDI suspensions: **(A)** temperature evolution as a function of time (0–400 s) under laser radiation by an 808 nm laser at a power of 1.8 W/cm^2^. PDI (0.04 mg/mL) and PEI (150 mg/mL) in DMF were used as the blank control, PEI-PDI suspensions are of a molar ratio of PEI repeat unit/PDI equals 4.5, 18.1, 181.4, 453.6, 725.7, 1314.3, 18143.2, respectively; **(B)** the evolution of maximum temperature (∆T), photothermal conversion efficiency (η) and the yield of free radicals in the PEI-PDI suspensions as a function of the molar ratio of PEI repeat unit/PDI. The experiment was conducted in a glovebox.

On the basis of the PDI-PEI composites with varying free radical yields, we anticipated that the highest yield of PDI radicals could attain a more effective NIR photothermal conversion. Hence, photothermal conversion experiments were performed with an 808-nm laser radiation at 1.8 W/cm^2^ at room temperature (29.8°C). The temperature elevations of PDI-PEI suspensions containing varied amounts of PDI radicals were measured and are presented in Figure 4. A blank test demonstrated that the temperature of a pure high concentration of PEI (150 mg/mL) and of pure PDI (0.04 mg/mL) in DMF increased only by 0.9°C and 1.1°C, respectively, within 400 s, while a significant increase in temperature was observed after irradiating DMF suspension with doped PDI-PEI. The consistent dependence of the increased temperature (∆*T*) and the photothermal conversion efficiency ( η ) on the molar ratio of PEI repeat unit/PEI indicates a direct correlation between them and the PDI free radical yield. (The yield of radical anions was estimated from the absorption at 698 nm with the Beer–Lambert law, *A= ɛcL*, where *ɛ* is the molar absorptivity, *L* is the length of the light path, and *c* is the concentration of the radical anions; the highest yield was normalized to 100% as shown in Figure 4B). The PDI-PEI suspension containing a higher yield of PDI radicals had a greater temperature increase than the suspension containing a lower yield of PDI radicals. An increase of PDI dianions and PEI content does not result in obvious photothermal conversion under irradiation due to no UV-vis absorption in the NIR range. Within 400 s, the temperature of a PDI-PEI suspension with a PDI radical yield of 92.2% (molar ratio of PEI repeat unit/PDI = 181.4) increased by 14.8°C, corresponding to a photothermal conversion efficiency of 47.9% calculated from its cooling curve. Meanwhile, the PDI-PEI suspension with a PDI radical yield of 14.0% (molar ratio of PEI repeat unit/PDI = 4.5) had a temperature increase of 2.3°C and a photothermal conversion efficiency *η* = 23.7%. The temperature of a PDI-PEI suspension with a PDI radical yield of 37.2% (molar ratio of PEI repeat unit/PDI = 18,143.2) increased by 4.8°C, corresponding to *η* = 32.0%.

To summarize, the photothermal conversion efficiency was tuned in the range of 23.7% to 47.9% for the PDI-PEI suspensions that have different yields of radical anions generated by varying the amounts of PEI doping. Note that the highest photothermal conversion efficiency of the unsubstituted PDI radical anion (47.9%) is comparable to that of functionalized perylene diimide radical anion salts (54.4%) with tetraphenoxy moieties onto the PDI cores and diisopropylbenzene in the amine position (TPPDI) (10^−4^ M in DMF) (Li et al., 2019). The efficiency is larger than a bola-form amphiphile PDI derivative’s radical anions (BPDI radical anion, 16.3%) and the supramolecule composed of BPDI and cucurbit [n]urils (CB [7]) free radical anion (BPDI/(CB [7])_2_ radical anion, 31.6%) (Jiao et al., 2015). The efficient photothermal conversion exhibited in this benchmark PDI with the simplest molecular structure highlights the necessity to tune the PDI to explore novel properties of this fundamental PDI molecule.

## 4 Conclusion

In this work, PEI was employed as an effective n-doping agent for feasible and controllable doping of PDI to form radical anions and dianions. DLS and SEM results show that the twinning of PEI on PDI results in an irregular morphology and an increase in PDI diameter. UV spectroscopy reveals the presence of PEI hindered the aggregation of the neutral PDI monomer. The stability of radical anions was improved compared to imide-position-substituted derivatives, although it falls behind its bay-stable imide-position-substituted derivatives. The radical-rich PDI-PEI suspensions presented tunable NIR photothermal conversion properties (23.7 %–47.9%). This research provides a new strategy to tune the doping level of similar n-type molecules for varying yields of radical anions and also illuminates the control of radical anion-based photothermal conversion efficiency.

## Data Availability

The original contributions presented in the study are included in the article/Supplementary Material; further inquiries can be directed to the corresponding author.

## Supplementary material

SUPPLEMENTARY FIGURE S1
*In situ* UV-vis spectra of PDI at time = 0 s (black curve) and 500 s (red curve) under constant voltage. Pristine PDI (∼10–4 M) in DMF with 0.1 M Bu_4_NPF_6_ was used as the electrolyte, a constant voltage of −1.8 V (which is enough to reduce PDI to get radical anion and dianion in a relatively short time) was applied at the Pt working electrode, the counter electrode is Pt and the reference electrode is Ag/Ag^+^.

SUPPLEMENTARY FIGURE S2 Dynamic Light Scattering result of the PDI-PEI suspension.

SUPPLEMENTARY FIGURE S3 g-factor of PDI suspension doped with PEI content under a molar ratio of PEI repeat unit to PDI equals to 9.1, 453.6, and 907.2.

SUPPLEMENTARY FIGURE S4
**(A)** M-H hysteresis loops measured four times (1st: one day; 2nd: 13 days; 3rd: 22 days; 4th: one month) for the sample molar ratio of PEI repeat unit/PDI = 453.6; **(B)** ferromagnetic contribution extracted from Supplementary Figure S4A.

SUPPLEMENTARY FIGURE S5 LUMO-HOMO energy of PDI with or without PEI: **(A)** Linear sweep voltammograms obtained in the glovebox of PDI aggregation in DMF without PEI (black), with various amounts of PEI (red) and the PDI monomer produced by oxidizing the doped PDI solution with air (blue); **(B)** UV-vis spectra of PDI aggregation in DMF with or without PEI used for the calculation of the energy gap between their HOMO and LUMO. The PDI suspensions in DMF with or without PEI are sonicated before cyclic voltammetry, PDI concentration: 0.05 mg/mL, the numbers in the brackets represent the PEI/PDI mass ratio 0, 1, 10, 50, 100, and 1000. Scan rate: 100 mV/s, working electrode: glassy carbon (Diameter = 3 mm); The LUMO energy for oxygen and water reduction was estimated from onset reduction potential from **(C)** linear sweep voltammograms of DMF electrolyte with saturated O_2_ or with deoxygenated H_2_O (glassy carbon as working electrode, scan rate:100 mV/s).
