# Supplementary material for: Chemical doping of unsubstituted perylene diimide to create radical anions with enhanced stability and tunable photothermal conversion efficiency
Source: Front Chem. 2023 Apr 25;11:1187378. doi: 10.3389/fchem.2023.1187378 (PMC10166849; doi:10.3389/fchem.2023.1187378)
Supplement: Supplementary file 1 [file DataSheet1.docx]

***Supplementary Material***

Chemical doping of unsubstituted perylene diimide to create radical anions with enhanced stability and tunable photothermal conversion efficiency

**Canyan Che^1#,^ Shaohua Tong^1#^, Yanhua Jia^1^, Jiaji Yang^1^, Xiandong He^1^, Shaobo Han^2^, Qinglin Jiang^1^,** **Yuguang Ma^1*^**

^1^State Key Laboratory of Luminescent Materials and Devices, Institute of Polymer Optoelectronic Materials and Devices, South China University of Technology Guangzhou 510640, China.

^2^School of Textile Materials and Engineering, Wuyi University, Jiangmen 529020, China.

***Correspondence:** Yuguang Ma**:** [ygma@scut.edu.cn](mailto:ygma@scut.edu.cn).

^#^These authors contributed equally.


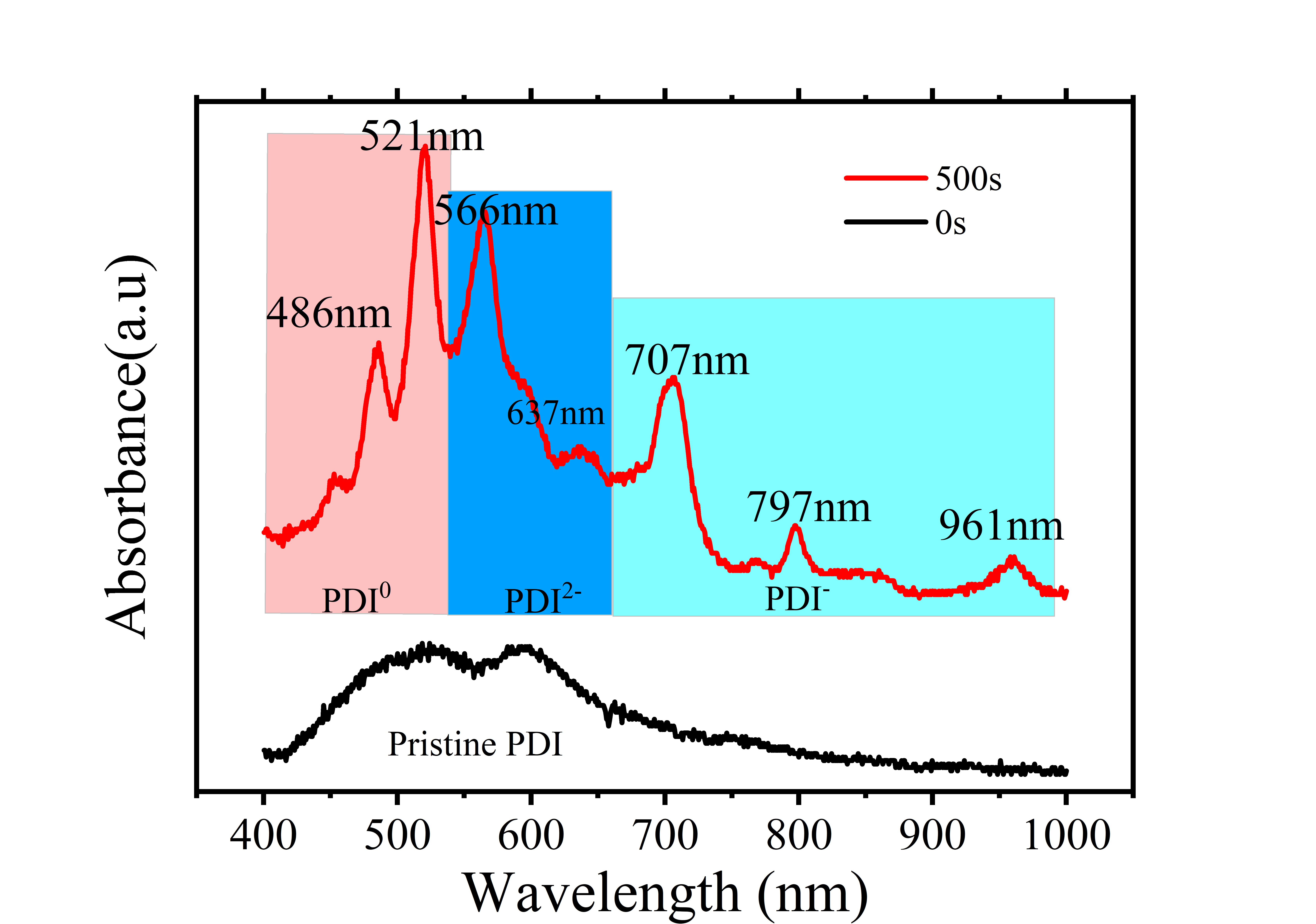


Supplementary Figure S1. In situ UV-vis spectra of PDI at time=0 s (black curve) and 500 s (red curve) under constant voltage. Pristine PDI (∼10^-4^ M) in DMF with 0.1M Bu_4_NPF_6_ was used as the electrolyte, a constant voltage of -1.8 V (which is enough to reduce PDI to get radical anion and dianion in a relatively short time) was applied at the Pt working electrode, the counter electrode is Pt and the reference electrode is Ag/Ag^+^.


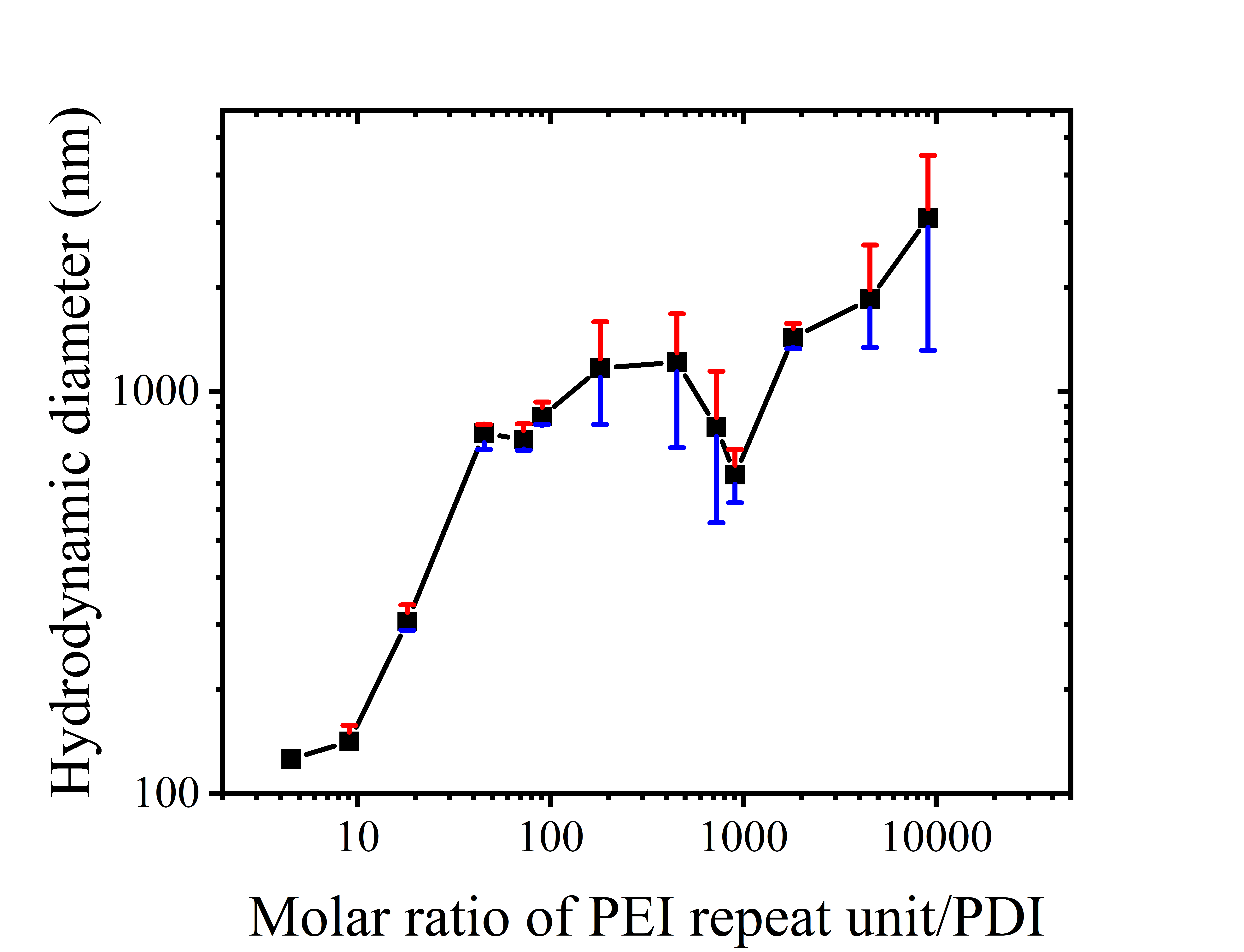


Supplementary Figure S2. Dynamic Light Scattering result of the PDI-PEI suspension.


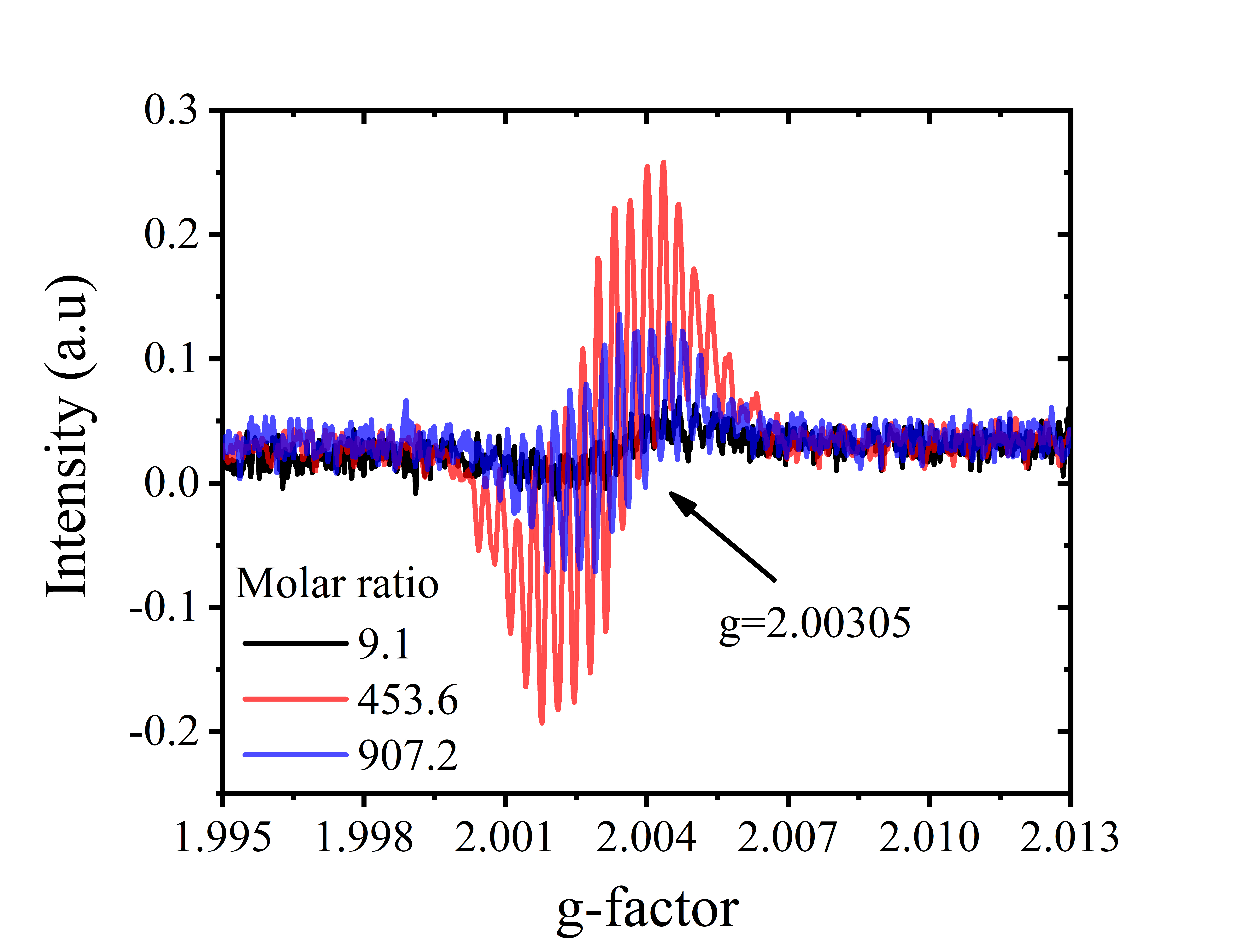


Supplementary Figure S3. g-factor of PDI suspension doped with PEI content under a molar ratio of PEI repeat unit to PDI equals to 9.1, 453.6, and 907.2.


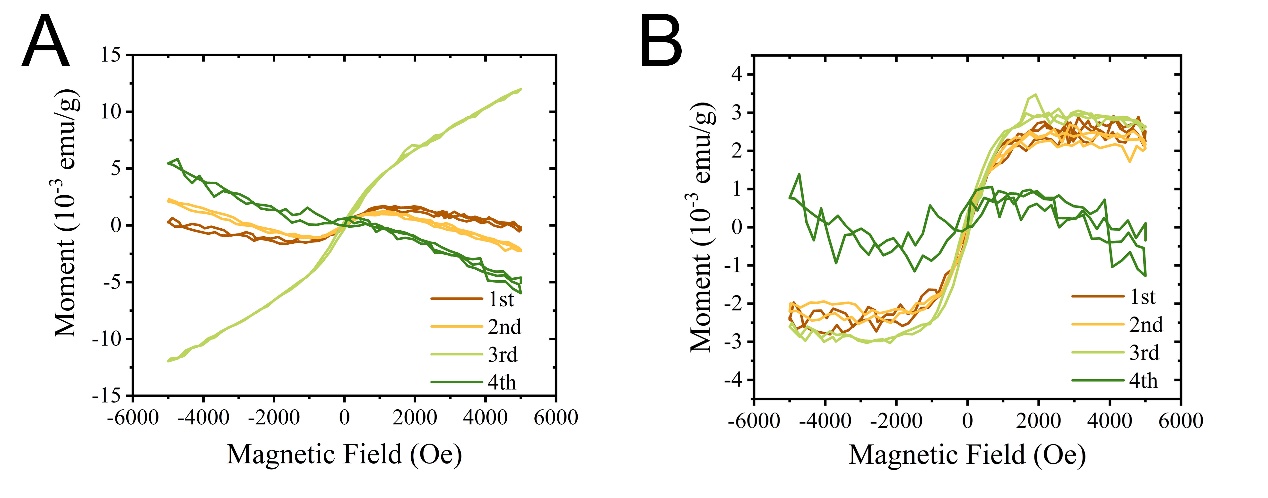


Supplementary Figure S4. A) M-H hysteresis loops measured four times (1^st^: one day; 2^nd^: 13days; 3^rd^: 22days; 4^th^: one month) for the sample molar ratio of PEI repeat unit/PDI=453.6; B) the ferromagnetic contribution extracted from Supplementary Figure S4A.

Ferromagnetism, or net spin and spin magnetic moment, could be present when electron spins in paramagnetic free radicals interact intensively and form an ordered orientation. On the base of free radicals obtained by varying amounts of PEI, we carried magnetic measurements on the drop-casted films of PDI-PEI suspensions with radicals accounting for the majority. The suspensions were dropped on Filter Paper and encapsulated by a laminator before magnetic measurement.

As shown in Supplementary Figure S4A, hybrid magnetism (dia-ferromagnetism for 1^st^, 2^nd^, ferro-para magnetism for 3^rd^ measurement) to diamagnetism (4^th^ measurement) was observed on the same sample, and it may be caused by oxidation PDI dianions and radical anions with trace amounts of oxygen getting in when the encapsulated paper was folded and squeezed during each measurement, which evidenced the significance to control and stabilize the doping level for the formation of ferromagnetic PDI. The ferromagnetic contribution in hybrid magnetism is 3*10^-3^ emu/g (Supplementary Figure S4B). The net contribution of PDI radical anion on in the composite (mass ratio of PEI/PDI=50) would be about 0.153 emu/g, it is only about 12.8% of that reported on the highly aggregated PDI radical anion without PEI (1.2emu/g, obtained by using hydrazine hydrate as reducing agent and spontaneously oxidation in the air). The one lower value of saturated magnetism was deduced as proof of the suppressed aggregation or loose π-π stacking of PDI radicals in presence of PEI.


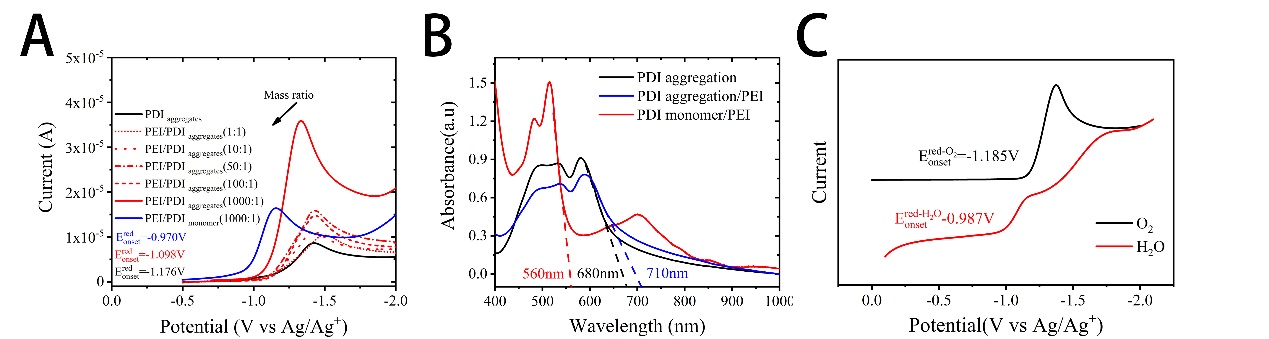


Supplementary Figure S5. LUMO-HOMO energy of PDI with or without PEI: A) Linear sweep voltammograms obtained in the glovebox of PDI aggregation in DMF without PEI (black), with various amounts of PEI (red) and the PDI monomer produced by oxidizing the doped PDI solution with air(blue); B) UV-vis spectra of PDI aggregation in DMF with or without PEI used for the calculation of the energy gap between their HOMO and LUMO. The PDI suspensions in DMF with or without PEI are sonicated before cyclic voltammetry, PDI concentration: 0.05 mg/mL, the numbers in the brackets represent the PEI/PDI mass ratio 0, 1, 10, 50, 100, and 1000. Scan rate: 100 mV/s, working electrode: glassy carbon (Diameter=3 mm); The LUMO energy for oxygen and water reduction was estimated from onset reduction potential from C) linear sweep voltammograms of DMF electrolyte with saturated O_2_ or with deoxygenated H_2_O (glassy carbon as working electrode, scan rate:100 mV/s).

Energy gaps were calculated from UV/vis absorption spectra according to ∆E=1240 nm/λ_onset_, where λ_onset_ is long wavelength edge of the absorption band, so it is 1.824 eV for PDI aggregation, 1.746eV for PDI aggregation with PEI, and is about 2.214 eV for PDI monomer with PEI.

The LUMO energy of water and oxygen in DMF is estimated with:

$$H_{2}O+e^{-}\to\frac{1}{2}H_{2}+OH^{-}, E_{onset}^{red}=-0.987V$$

E(LUMO)=-(4.8-0.9870-0.089) = -3.724eV

-1.185V


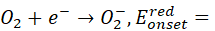

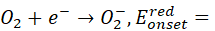


E(LUMO)=-(4.8-1.185-0.089) = -3.526eV
